# Disability status and quality of life in multiple sclerosis: non-linearity of the Expanded Disability Status Scale (EDSS)

**DOI:** 10.1186/1477-7525-8-55

**Published:** 2010-06-07

**Authors:** Sabine Twork, Susanne Wiesmeth, Milena Spindler, Markus Wirtz, Sabine Schipper, Dieter Pöhlau, Jörg Klewer, Joachim Kugler

**Affiliations:** 1 Faculty of Medicine at the University of Technology Dresden, Department Health Sciences/Public Health, 01307 Dresden, Germany; 2 Department of Medical Psychology, University Clinic of Essen, 45122 Essen, Germany; 3 Department of Neurology, Kamillus Hospital, 53567 Asbach, Germany

## Abstract

**Background:**

Progression in disability as measured by increase in the Expanded Disability Status Scale (EDSS) is commonly used as outcome variable in clinical trials concerning multiple sclerosis (MS). In this study, we addressed the question, whether there is a linear relationship between disability status and health related quality of life (HRQOL) in MS.

**Methods:**

7305 MS patients were sent a questionnaire containing a German version of the "Multiple Sclerosis Quality of Life (MSQOL)-54" and an assessment of self-reported disability status analogous to the EDSS. 3157 patients participated in the study. Patients were allocated to three groups according to disability status.

**Results:**

Regarding the physical health composite and the mental health composite as well as most MSQOL-54 subscales, the differences between EDSS 4.5-6.5 and EDSS >= 7 were clearly smaller than the differences between EDSS <= 4 and EDSS 4.5-6.5.

**Conclusion:**

These results indicate a non-linear relationship between disability status and HRQOL in MS. The EDSS does not seem to be interval scaled as is commonly assumed. Consequently, absolute increase in EDSS does not seem to be a suitable outcome variable in MS studies.

## Introduction

Progression in disability as measured by increase in the Expanded Disability Status Scale (EDSS) is a frequently used outcome variable in clinical trials concerning multiple sclerosis (MS) [1-5].

However, EDSS represents only a part of health related quality of life (HRQOL) in MS. HRQOL comprises several domains in addition to physical impairments like social functioning and psychological well-being [6,7]. There is a growing interest in HRQOL in MS patients and several studies have been performed addressing this topic [8-18]. MS-patients have reduced quality of life within the different aspects of HRQOL in comparison to the general population [17,19-23]. There appears to be a relationship between EDSS and HRQOL, although some studies find a relationship only with physical functioning, rather with psychological functioning or psychological well-being [9,11,22,24-29].

Results from several studies suggest a non-linear relationship between EDSS and HRQOL in MS. In a study by Vickrey et al., the difference between patients who walked with an aid and wheelchair-bound patients was smaller than the difference between patients walking without help and patients being dependent on a walking aid in most HRQOL domains [30]. Similarly, Patti et al. reported no significant difference between a patient group with high EDSS scores and a group with moderate EDSS scores in nearly all domains, whereas groups with low and moderate EDSS scores differed significantly in all domains [26].

In this study, comprising a large sample with a broad spectrum of patients, we addressed the question, whether there is a linear relationship between disability status and HRQOL in patients with MS. We therefore explored the difference regarding HRQOL between patients with differing ambulation status. Three groups of patients were compared for this purpose: patients who are confined to a wheelchair, patients who are impaired in ambulation without being wheelchair-bound, and patients with largely unimpaired ambulation.

## Materials and methods

### Sample

Data were collected using a postal survey of all 7305 MS-patients registered as patient members of the German MS Society in North Rhine-Westphalia. 3157 patients volunteered to participate, giving a response rate of 43.2%.

### Questionnaires

The survey included a German version of the Multiple Sclerosis Quality of Life (MSQOL)-54 Questionnaire and a structured demographic and clinical questionnaire [30].

The MSQOL-54 comprises questions from the Short Form 36-Item Health Survey as a generic measure of quality of life, and 18 additional MS specific items [7,31,32]. The MSQOL-54 has high test-retest reliability and high internal consistency. Evidence supports its content and construct validity [30,33]. Persian, Finnish, Greek, and Serbian versions have recently been validated [34-37], as well as Italian, French and Turkish versions have been validated a few years ago [38-40]. There is evidence that also the German version used here has satisfactory reliability and validity [41]. The 54 items of the MSQOL-54 are distributed into 12 multi-item scales and two single-item scales [30]. Due to technical and psychometric reasons the scale "vitality" was reduced by one item. For statistical analysis scale scores were created by averaging items within scales and transforming average scores linearly to 0-100 possible scores, with higher values indicating better quality of life. Missing values were replaced by the mean of the remaining items constituting a scale, unless there were missing more than 50% of items in the concerning scale. In addition physical and mental health composite scores were determined as weighted sums of selected scale scores as described by Vickrey et al. [30]. Composite scores, too, had a range of 0-100.

Self-report questions about ambulation status were developed which were analogous to information from the Expanded Disability Status Scale (EDSS) [42]. The EDSS has a possible range from 0, indicating no disability and normal neurological examination, to 10, referring to death due to MS. For this analysis, patients were allocated to one of three groups based on their responses to the self-report questions. Group 1 included patients who were able to walk at least 1000 meters without help (corresponding to an EDSS score <= 4.0). Group 2 comprised people able to walk at least 100 metres with or without aid (EDSS score between 4.5 and 6.5). Patients in group 3 were largely confined to a wheelchair (EDSS >= 7.0).

Duration of disease was expressed as years of time since diagnosis instead of time since onset of disease.

### Procedures

In July and August 2004, patients were sent the questionnaires and a cover letter which asked for participation, explained the importance of participation and clearly stated that all information would be treated in strict confidence. A pre-stamped and pre-addressed envelope was included. Patients were invited to call if they required further information.

### Statistical Analysis

Univariate analyses of covariance (general linear model) were computed to evaluate differences regarding MSQOL subscales and composite scores between subgroups with different ambulation status. Gender was included as an additional factor while age and duration of disease were considered as covariates.

Due to multiple comparisons the results were considered statistically significant only if p values were <.01.

Statistical analysis was performed with the Statistical Package for Social Sciences (SPSS), version 15.

The research protocol of the study was reviewed and approved by the Committee of Research Ethics at the Medical Faculty of the Technical University of Dresden. It was carried out in accordance with the Declaration of Helsinki. All subjects received written information on the study and gave written informed consent prior to participation.

## Results

Proportions of missing results varied between 3.8% and 7.8% for the mentioned demographic and disease-related variables and between 1.9% (cognitive function) and 16.5% (sexual function) for MSQOL-54 subscales after replacing missing values as described above.

### Demographic and disease related characteristics

71.7% of the 3045 respondents were women (table 1). The mean age of the respondents was 48.2 years. Mean duration of disease was 13.2 years. 1429 (47.6%) patients were able to walk at least 1000 metres without help (group 1), 633 (21.1%) patients could walk at least 100 metres (group 2), and 938 (31.3%) patients were largely confined to a wheelchair (group 3). The three groups differed clearly from each other in terms of gender, age, and duration of disease, i.e., the worse the ambulation status, the higher the proportion of men and the higher age and disease duration.

**Table 1:** Demographic and disease characteristics of the total sample and of the three ambulation status subgroups

		Ambulation status		
		
	Total sample	Group 1	Group 2	Group 3
n	3157	1429	633	938
% Women	71.7	77.0	70.5	63.6
Age (years; m +/- SD)	48.2 +/- 11.8	42.9 +/- 9.8	50.4 +/- 10.5	54.7 +/- 11.5
Years since diagnosis (m +/- SD)	13.2 +/- 9.4	9.3 +/- 7.1	13.8 +/- 9.0	18.6 +/- 9.8

### Quality of Life

The scores of both MSQOL-54 composites and most subscales were distributed over a broad spectrum. Only in the role limitations (physical) scale and the role limitations (emotional) scale, there were so called floor and ceiling effects (i.e., many very low or very high scores).

**Figure 1:**
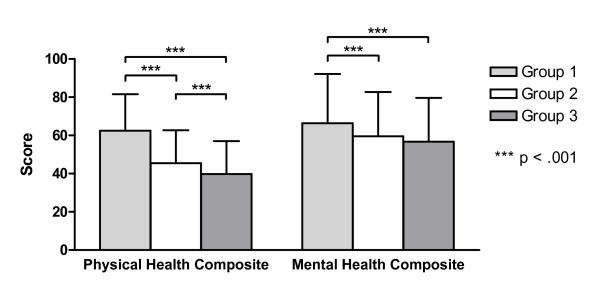
**Mean MSQOL-54 composite scores (adjusted for effects of age and disease duration; with standard deviations) of the three ambulation status subgroups**.

As shown in figure 1, the physical health composite as well as the mental health composite score of the MSQOL-54 decreased significantly as a function of ambulation status (p = .000). The differences between groups 1 and 2 were clearly larger than those between groups 2 and 3. With regard to the physical health composite, group 1 and group 2 differed significantly from each other (p = .000) as well as groups 1 and 3 (p = .000) and groups 2 and 3 (p = .000). The mean scores for the mental health composite differed significantly only between group 1 and group 2 (p = .000) as well as between groups 1 and 3 (p = .000). The difference between groups 2 and 3 was not significant (p = .134).

The mean subscale scores of the MSQOL-54 are presented in table 2. The mean scores on all scales decreased with worsening ambulation status. The difference between groups 2 and 3 was smaller than the one between groups 1 and 2 on all of these subscales except for cognitive function. Groups 1 and 2 differed significantly with regard to all subscales (all p < .010) but cognitive function (p = 1.000) and role limitations (emotional) (p = .017). Between group 2 and group 3, significant differences were found only in physical function, social function, overall quality of life and sexual function (all p < .010). Regarding the other scales groups 2 and 3 did not differ significantly (p > .016 for pain and p > .055 for the remaining scales, respectively).

**Table 2:** Mean scores on the 12 MSQOL-54 subscales and the two composite scores for the total sample, by gender and by ambulation status (the latter two adjusted for effects of age and disease duration)

		Gender		Level of ambulation		
		
	Total Sample	Men	Women	Group 1	Group 2	Group 3
Physical function	44.0 +/- 33.9	37.7 +/- 22.1	37.1 +/- 23.2	67.9 +/- 26.1^b,c^	28.9 +/- 23.4^b,d^	15.4 +/- 23.3^d,c^
Role (physical)	35.8 +/- 40.6	35.0 +/- 37.9 ^a^	30.0 +/- 40.2 ^a^	51.2 +/- 44.9^b,c^	25.5 +/- 40.3^b^	20.9 +/- 40.1^c^
Role (emotional)	58.2 +/- 44.5	58.8 +/- 45.4	57.0 +/- 48.6	64.6 +/- 53.6^c^	57.1 +/- 48.0	52.1 +/- 47.5^c^
Pain	65.4 +/- 29.2	69.9 +/- 28.4 ^a^	62.5 +/- 29.6 ^a^	72.2 +/- 33.7^b,c^	65.5 +/- 30.0^b^	60.8 +/- 30.4^c^
Emotional well-being	61.3 +/- 20.1	62.1 +/- 21.2	60.6 +/- 22.0	64.0 +/- 25.0^b,c^	60.2 +/- 22.4^b^	59.9 +/- 22.5^c^
Energy	39.7 +/- 19.8	39.7 +/- 20.3	38.3 +/- 21.1	44.9 +/- 24.1^b,c^	37.0 +/- 21.6^b^	35.1 +/- 21.7^c^
Health perceptions	43.9 +/- 18.9	41.4 +/- 19.0	43.2 +/- 19.8	48.9 +/- 22.5^b,c^	40.2 +/- 20.1^b^	37.9 +/- 20.3^c^
Social function	65.1 +/- 24.9	63.0 +/- 23.5	63.7 +/- 24.4	75.2 +/- 27.8^b,c^	61.0 +/- 24.7^b,d^	53.8 +/- 25.1^d,c^
Cognitive function	68.4 +/- 22.9	66.5 +/- 24.0	69.1 +/- 24.9	69.0 +/- 28.4	68.2 +/- 25.4	66.3 +/- 25.7
Health distress	62.8 +/- 21.9	61.7 +/- 22.2	61.7 +/- 23.1	69.6 +/- 26.3^b,c^	59.3 +/- 23.5^b^	56.3 +/- 23.7^c^
Overall quality of life	58.0 +/- 24.5	55.0 +/- 23.0	56.2 +/- 24.0	66.8 +/- 27.2^b,c^	53.9 +/- 24.3^b,d^	46.1 +/- 24.6^d,c^
Sexual function	61.2 +/- 33.6	52.6 +/- 31.6 ^a^	65.0 +/- 33.2 ^a^	68.1 +/- 35.9^b,c^	57.7 +/- 32.3^b,d^	50.6 +/- 32.6^d,c^
physical health composite	52.7 +/- 19.7	49.6 +/- 16.9	48.9 +/- 18.3	62.5 +/- 19.1^b,c^	45.5 +/- 17.2^b,d^	39.8 +/- 17.2^d,c^
mental health composite	62.0 +/- 21.3	61.1 +/- 22.0	60.6 +/- 23.6	66.3 +/- 25.8^b,c^	59.6 +/- 23.1^b^	56.7 +/- 22.9^c^

^a^p < .01 men vs. women (ANCOVA)

^b^p < .01 group 1 vs. group 2 (ANCOVA)

^c^p < .01 group 1 vs. group 3 (ANCOVA)

^d^p < .01 group 2 vs. group 3 (ANCOVA)

Men and women differed significantly in role limitations (physical), pain and sexual function (all p < .010), with men having the higher score in pain and the lower scores in role limitations (physical) as well as sexual function (table 2). Gender effects were not significant on the other subscales and on the composite scores (p > .014 for health perceptions and cognitive function, and p > .110 for the remaining scales, respectively).

**Figure 2:**
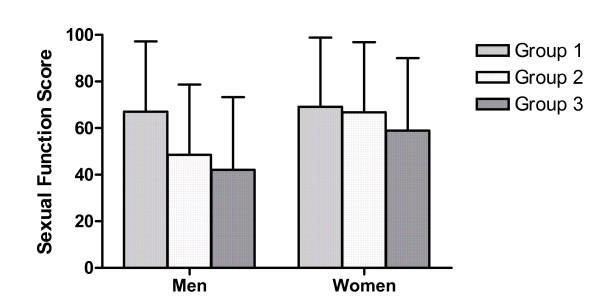
**Mean sexual function scores (adjusted for effects of age and disease duration; with standard deviations) of the three ambulation status subgroups for men and for women**.

Regarding sexual function, there was a significant interaction effect between gender and ambulation status subgroup (p = .000). As can be seen in figure 2, in men the difference between groups 1 and 2 (mean 67.1 and 48.5, respectively) was clearly larger than the difference between group 2 and 3 (42.1), whereas in women groups 1 and 2 differed only slightly (69.1 and 66.8, respectively) in comparison to group 2 and group 3 (59.0). Regarding the other scales, no significant interaction effects were found (p = .028 for health distress and p > .092 for the remaining scales, respectively).

## Discussion

The goal of the study was to explore the nature of the relationship between disability as measured by EDSS and HRQOL in MS. Therefore, we assessed the differences between patient groups with differing disability status regarding their MSQOL-54 scale scores.

The physical and the mental health composite of the MSQOL-54 as well as all subscale scores but cognitive function decreased with worsening disability status. However, while patients with an estimated EDSS score between 4.5 and 6.5 differed markedly from patients with a lower EDSS (0 - 4.0), the difference between the two groups of patients with higher EDSS scores (4.5 - 6.5 and 7.0 - 10.0, respectively) was smaller on all of these scales, not even being significant on the mental health composite and most of the subscales. Hence, the results suggest, that whereas impaired ambulation (without confinement to a wheelchair) is accompanied by striking deteriorations in HRQOL, being bound to a wheelchair does not lead to decisive additional decreases in most HRQOL domains.

The results are in agreement with those of previous studies. In a study by Patti et al. patients with EDSS scores lower 3.0 scored significantly better than two groups with higher EDSS scores in all dimensions of the SF-36 [26]. In the contrary, there was less difference between these latter two groups. Patients with EDSS scores of 3.0 to 6.0 had markedly higher scores than patients with EDSS scores over 6.0 only for physical functioning. Vickrey et al., too, found larger differences between patients who could walk without help and patients who required assistance to ambulate than between this latter group and patients who were confined to a wheelchair on most MSQOL-54 scales [30].

Interestingly, in our study this pattern was found for sexual function only in men. In women the difference between groups 1 and 2 was smaller than in men, whereas the difference between groups 2 and 3 did not differ from the one found in men. Hence, the relationship between ambulation status and sexual dysfunction seems to be different in men and in women.

Consistently with the results of other studies, cognitive function scores did not vary significantly between groups [43,44]. Hence, cognitive function and ambulation appear to be independent.

Gender effects were only observed in three subscales. Likewise, other studies reported gender effects in only few or none of the HRQOL domains, respectively [17,21,22,26,44-46].

The data obtained in this study clearly suggest a non-linear relationship between HRQOL and disability status as measured by EDSS. This is the case for physical as well as for mental quality of life. The EDSS does not seem to be interval scaled as is commonly assumed. The step from largely unimpaired ambulation to impaired ambulation status and the next step towards restriction to a wheelchair are not equal in distance. Therefore, absolute increase in EDSS, e.g. one point per year, is not suited for outcome variable in clinical trials in which the effectiveness of MS immunotherapy is examined. This outcome measure has been used in a number of decisive clinical trials [1-3]. Following the findings of the present study, this is inappropriate, unless the investigators take care of strictly homogenous samples with low variations in baseline disability status. As an alternative, a proportional decrease in disability as assessed relative to the baseline EDSS scores should be used as an outcome measure. In general, MS-specific measure of quality of life should be used for detecting treatment effects [47]. Future research may address the precise definition of such a measure.

Assessing EDSS scores based on self-ratings of ambulation status can be seen as one limitation of this study. Yet, the EDSS itself relies heavily on ambulation. Besides, a number of authors emphasize that a self-rating of disability status can be seen as a good substitute for neurological assessment if this is not available [25,26,48]. The advantage of this proceeding in the present study was that it was possible to examine a very large sample this way.

The German MS Society is divided into parts similar to the federal states of Germany and supports around 50,000 MS patients. The patients from the North Rhine-Westphalian section represent the largest proportion compared to the other German sections. The question is to which extent these patients are representative for the whole German MS community. On the other hand a possible selection bias has to be discussed. Thus, members of the MS Society could form a special subgroup (maybe older, with longer disease duration) and patients working on the questionnaire could be especially motivated. The response rate obtained in this study was not as high as was hoped for, but the sample nonetheless is relatively large and covers a brought spectrum of patients. With regard to age, gender and duration of disease the distribution given in this sample is in line with the one found in other studies of MS patients [9,22,45,46,49].

## Conclusion

In conclusion, the results of the present study indicate a nonlinear relationship between disability status and HRQOL. They therefore contradict the assumption that the EDSS is interval scaled. Consequently, absolute increase in EDSS does not seem to be a suitable outcome variable in MS studies.

## Competing interests

The survey was funded by the German MS Society "Deutsche Multiple Sklerose Gesellschaft" (DMSG) in North Rhine-Westphalia, Germany.

There are no redundant publications or conflicts of interest.

## Authors' contributions

ST and SW performed the statistical analysis and drafted the manuscript. MS helped to draft the manuscript. MW conceived the study and drafted its design. SS helped to conceive the study and participated in the data collection. DP gave advise and helped to draft the manuscript. JKl developed the design of the questionnaires, helped to conceive the study and managed the project. JKu conceived the study, drafted its design and helped to manage the project.

All authors read and approved the final manuscript.
